# Mapping roof coverings of asbestos-cement, the first step to control the technical condition/threat and establish priorities for replacement in developing countries

**DOI:** 10.1016/j.heliyon.2024.e37522

**Published:** 2024-09-06

**Authors:** Leydy K.Torres Gil, David Valdelamar Martínez, Kellys Babilonia Franco, Alfonso Arrieta Pastrana, Manuel Saba

**Affiliations:** Civil Engineering Program, Universidad de Cartagena, Calle 30 #, 48-152, Cartagena, Colombia

**Keywords:** Multi-criteria assessment, Asbestos weathering status index, Priority index

## Abstract

This paper presents a systematic and data-driven approach to prioritize interventions in urban areas with asbestos cement (AC) roofs, addressing the urgent need to mitigate asbestos-related risks. The objective is to propose a comprehensive methodology that considers multiple criteria at the neighborhood level, allowing for a nuanced assessment of intervention priorities.

The methodology involves the normalization of various parameters, including population density, facility density, and the area covered by asbestos-cement roofs. In addition, an innovative aspect is introduced by incorporating weathering status identification data, represented as an index, validated in previous research, further enriching the evaluation process. The integration of these diverse factors allows for a holistic understanding of the risk landscape associated with AC roofs in urban settings.

The cornerstone of the proposed approach is the development of a Priority Intervention Index (PII) at the neighborhood level. This index serves to standardize the assessment of intervention priorities, enabling a fair and transparent comparison across different regions. To enhance practical application, the PII is discretized into three categories, low, mid and high intervention priority. The results obtained are robust, replicable in other scenarios, and practical for decision-makers. The new methodology provides a structured and quantifiable approach to identify and prioritize areas for asbestos-related interventions based on well-defined criteria at the neighborhood level. The resulting prioritization strategy offers urban planners and local officials a clear and evidence-based tool to allocate resources efficiently and effectively manage the inherent risks associated with AC roofs in urban environments. The paper will describe how the prioritization can be applied “at the neighborhood level” by urban planners and local officials.

## Highlights

• Systematic methodology considers population, facilities, and weathering in prioritizing AC roof interventions.
• Integration of an Asbestos Weathering Status Index enhances risk evaluation in urban areas.
• Cross-regional comparisons for decision-makers on asbestos related problems.
• Quantifiable tool for managing asbestos-related risks in urban environments.

## 1 Introduction

Asbestos, a group of naturally occurring minerals renowned for its resistance to heat and durability [1,2], has long been a cornerstone of various industrial applications and construction materials [[3], [4], [5], [6], [7], [8]]. Among its many uses, asbestos-cement (AC) roofs have become a ubiquitous feature in urban landscapes worldwide, owing to the material's resilience and cost-effectiveness in construction [9]. However, the seemingly innocuous presence of asbestos conceals a latent threat to public health, particularly when these roofing materials undergo degradation or disturbance, potentially releasing harmful asbestos fibers into the surrounding environment [5,10,11].

Health hazards associated with asbestos exposure have been well-documented in scientific literature [[12], [13], [14], [15], [16], [17], [18], [19], [20], [21]]. On the other hand, regarding mitigation measures, the focus has traditionally leaned towards the identification and quantification of asbestos-containing material through remote sensing [[22], [23], [24], [25]]. Recognizing asbestos-cement roofs is a crucial step in understanding the extent of potential exposure. Numerous studies have delved into the development of remote sensing and spectral analysis techniques for identifying these roofs, employing advanced technologies to map and monitor areas with asbestos-related risks [26,27]. However, a critical gap in the existing body of literature becomes apparent when one examines the limited attention given to the prioritization of interventions to mitigate the risks associated with exposure to asbestos. Despite the wealth of research devoted to the spectral characterization of AC roofs, only a handful of works extend their inquiry into the realm of defining intervention priorities [25,28]. This gap in the literature is particularly pronounced when considering the multifaceted nature of risk factors associated with asbestos exposure in urban areas.

The prevailing trend in existing studies predominantly fixates on the spectral response of AC roofs, neglecting a broader set of criteria that could significantly contribute to the development of a comprehensive intervention prioritization framework. Parameters such as population density, social strata, and facility density are integral aspects that warrant consideration. These factors are crucial in capturing the complex dynamics of urban environments and assessing the vulnerability of communities to the risks posed by deteriorating asbestos-cement roofs. Other parameters such as the individual's possible exposure to asbestos in the workplace or the presence of comorbidities, although they would be very useful to define intervention criteria, are information that is generally not available or difficult to obtain in large scale.

The dearth of studies addressing these multifactorial criteria underscores the pressing need for a holistic and systematic approach to prioritize interventions. The challenge lies not only in the identification of AC roofs but, more critically, in establishing a well-defined and comprehensive system for prioritizing interventions based on multiple criteria. Such a system is especially vital in developing countries that are grappling with asbestos-related problems. In developed nations, substantial financial incentives have been offered in recent decades to reduce the public health risks associated with asbestos. However, in developing counties this strategy cannot be applied since these regions often face economic constraints, necessitating the optimization of available resources for targeted and impactful interventions.

In Colombia, the legal status of asbestos products underwent a significant change with the enactment of Law 1968 of 2019, which instituted a complete ban on asbestos starting from January 2021. However, the regulatory landscape remains limited, with no comprehensive framework beyond the prohibition itself. At present, there are no established plans for asbestos removal, allocation of funds, or public awareness campaigns regarding this issue. This situation parallels the circumstances of developing countries from several decades ago, where asbestos regulation was similarly minimal. In contrast to more developed nations [29], which have implemented stringent regulations and comprehensive programs for asbestos management and removal, Colombia's approach appears less proactive. The absence of mandatory registration, technical assessment, and disposal protocols for asbestos-containing products in buildings reflects a gap in the regulatory framework, underscoring the urgent need for prioritized action to address this pressing public health concern.

In this context, the present research seeks to bridge this gap by proposing a systematic and data-driven approach for prioritizing interventions in urban areas with asbestos-cement roofs.

The present manuscript constitutes a subsequent investigation stemming from a recently published study [30], wherein the authors delved into the assessment of weathering phenomena by juxtaposing on-site degradation evaluation against hyperspectral degradation identification. Despite methodological disparities between on-site and hyperspectral data analyses, two of the implemented methodologies evinced promising outcomes, underscoring their efficacy in delineating roofing structures exhibiting both satisfactory and compromised conditions on a macroscopic scale, as inferred from hyperspectral imagery.

The methodology outlined in this paper goes beyond the limitations of existing studies by incorporating a diverse range of criteria, from demographic factors to the condition of the roofs themselves, culminating in a comprehensive Priority Intervention Index (PII). This index provides a standardized and transparent means of assessing and communicating the urgency of interventions, thereby offering decision-makers a valuable tool at the neighborhood level to optimize resource allocation and guide public policies effectively. The significance of adopting a multidimensional approach to prioritize interventions becomes increasingly apparent, representing a pivotal step towards mitigating the risks associated with asbestos exposure in urban environments, especially in developing countries.

## 2 Methodology

The current research delineates its procedural framework in Fig. 1. Below, the authors expound upon the case study and the sequential stages of the methodology, dedicating a distinct section to each for comprehensive elucidation.

**Fig. 1:**
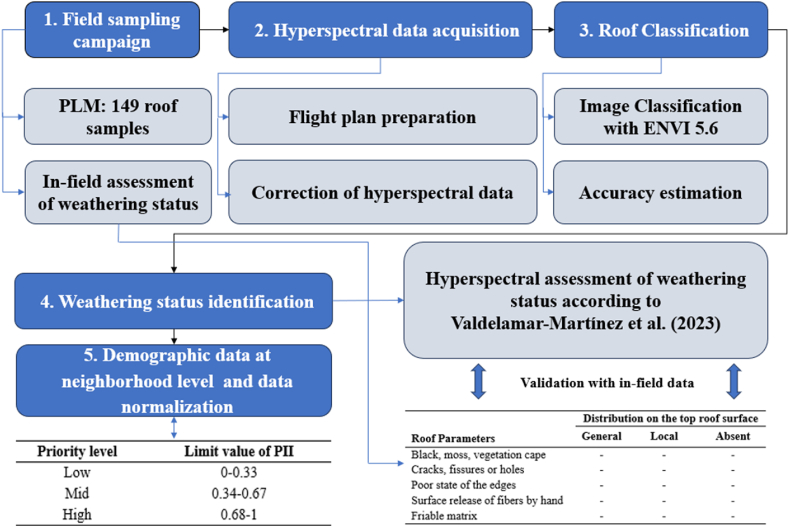
Methodology workflow of the present research.

### 2.1 Case of study and field sampling

The study concentrates on the urban area of Cartagena de Indias in northern Colombia, home to about one million people and covering an area of 80.9 km^2^, as depicted in Fig. 2. The urban milieu is characterized by a pronounced clustering of residential zones, a constrained prevalence of verdant spaces interspersed amidst the urban landscape, and a conspicuous dearth of cohesive urban planning, a scenario often encountered in emerging economies [31].

**Fig. 2:**
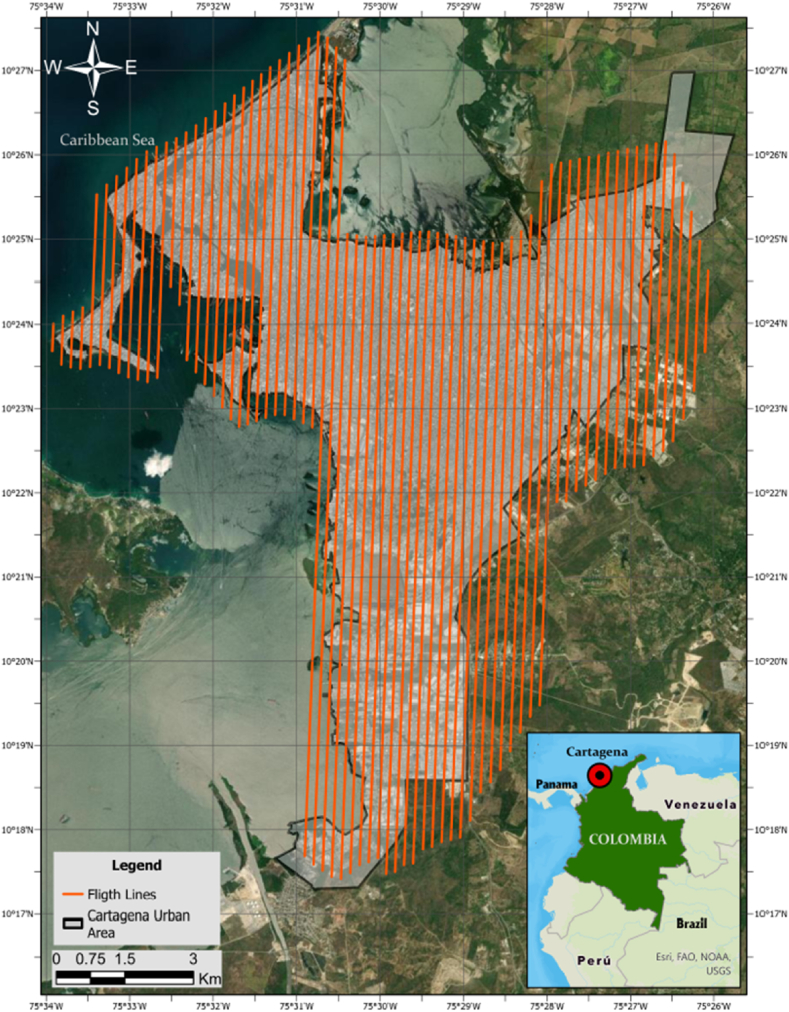
Location of the study area in Cartagena de Indias and flight plan.

During a field campaign, a total of 149 asbestos-cement (AC) roof samples, 1 cm × 1 cm, were randomly collected within the designated study area to identify the presence of asbestos fibres, following the *Polarized Light Microscopy (PLM) Validation Process Guidelines* of the U.S. Environmental Protection Agency (EPA), [32].

### 2.2 Hyperspectral data acquisition

Hyperspectral data collection was conducted through an aerial survey using the Mjolnir VS-620 sensor, developed by HySpex. This state-of-the-art sensor captures spectral data ranging from 400 nm to 2500 nm across 490 bands, with 200 bands in the visible range at approximately 3.0 nm spectral resolution and 290 bands in the short-wave infrared (SWIR) at around 5.1 nm resolution [33]. The ground sampling distance was 0.8 m.

The survey was carried out using a Cessna TU206F Turbo aircraft, which flew at an altitude of 800 m, as illustrated in Fig. 2. The Mjolnir sensor provided a swath width of 250 m per line of imaging, consistent with the flight plan. To ensure thorough coverage and minimize data gaps, a 25 % overlap between imaging lines was maintained. After data collection, the images were rigorously corrected with HySpex RAD for radiometric distortions, Rese PARGE for geometric errors, and Rese DROACOR for atmospheric disturbances.

### 2.3 Roof classification

Hyper-spectral data was processed using ENVI® 5.6 software. Noisy spectral bands were visually identified and removed to reduce computational complexity without compromising data integrity. The Minimum Noise Fraction (MNF) algorithm, a standard method for removing noise from hyper-spectral data, was used in this analysis. MNF transformed the noisy data into a series of output images, each with a different level of noise, as described in Ref. [26], and [34]. To simplify the analysis, a mask was created to identify urban areas, excluding roads, vegetation, and water bodies. Supervised classification was performed using the Spectral Angle Mapper (SAM) algorithm in ENVI®. Ten asbestos-cement (AC) roofs, confirmed to contain asbestos by PLM, were used as training samples.

These roofs were utilized to establish and refine the parameters of the SAM algorithm. Subsequently, the remaining 139 roofs contain asbestos were designated for validation. In this context, validation entails the rigorous assessment of the model's predictive performance and generalizability. These validation roofs serve as an independent dataset, distinct from the training set, against which the model's accuracy, reliability, and robustness are evaluated. Through this process, any biases or overfitting tendencies in the model are identified and rectified, ensuring its efficacy in accurately classifying asbestos-containing roofs beyond the confines of the training dataset.

The ENVI® software facilitates the classification of pixels corresponding to asbestos, thereby generating a distinct layer delineating these classified areas. Subsequently, leveraging the Geographic Information System (GIS) software, ArcGIS®, a sophisticated analytical tool known as the “Calculation of Geometric Properties” is employed to accurately quantify the total area occupied by asbestos-cement within the designated region. This process entails meticulous computation and analysis within the GIS framework, ensuring precise determination of the extent of asbestos presence across the spatial domain under investigation.

### 2.4 Weathering status identification

In prior investigations [30], the authors successfully delineated the weathering conditions that distinguish between Low Intervention Priority (LIP) and High Intervention Priority (HIP) and scenarios through in-site inspections vs hyperspectral image analysis. Similar results are reported in the literature [25]. These priority levels pertain to the imperative necessity, or conversely, the lack thereof, for immediate intervention in asbestos-cement roofs. Such interventions may encompass substantial measures like AC removal or more moderate approaches like enclosure or encapsulation [4,[35], [36], [37]]. The Index of Surface Deterioration (ISD) focuses on the reflectance at the 2327 nm absorption peak, a characteristic shared by chrysotile, cement, and asbestos-cement materials (1).

(1) $${ISD}_{CAP}=4.13\;R_{Sample2327}-0.458$$

herein, ISD is the Index of Surface Deterioration, CAP denotes the Chrysotile Absorption Peak, and R_Sample2327_ signifies the recorded reflectance at 2327 nm for the asbestos-cement sample. A typical value for R_Sample2327_ could be 0.250 (adim.), resulting in a ISD_CAP_ of 0.57 (adim.) with R_Sample2327_ always <1 and ISD_CAP_ always <3.67. For more details of equation (1) see Valdelamar-Martínez et al. (2023) [30]. This index is computed for each pixel within the hyperspectral image. Lastly, the quantity of asbestos-cement pixels within each neighborhood was calculated.

### 2.5 Demographic data at neighborhood level and data normalization

The population data for the 190 neighborhoods within the city of Cartagena were acquired from the database of the National Administrative Department of Statistics of Colombia (DANE, for its initials in Spanish), [38]. Neighborhood-level data is stratified by age groups (0–9; 10–19; 20–29; 30–39; 40–49; 50–59; 60–69; 70–79; 80–89; and >90 years) and gender (male and female). In the context of this investigation, the research encompassed the examination of the overall population density.

On the other hand, the term 'Facilities' encompasses all publicly and privately owned facilities with public access, including nurseries, schools, universities, hospitals, clinics, retirement homes, assisted living facilities, government buildings, places of worship, shopping centers and public parks. These spaces are distinguished by a significant level of public accessibility, enduring over prolonged periods.

The identification of such locations was based on secondary data provided by the Cartagena Municipal Government [39]. The density of these facilities at neighborhood-level was determined using ArcGIS®.

Parameter normalization refers to the process of adjusting or scaling a given parameter so that its value falls within a specific range or scale, typically with the aim of enhancing data comparability or interpretability. This includes techniques such as min-max normalization, where values are scaled to fit within a designated range (such as 0 to 1). Normalization proves particularly advantageous when dealing with data of varying scales or magnitudes, facilitating more meaningful and precise data comparison and analysis while mitigating the potential dominance of one variable over others due to disparities in their original scales.

Data normalization was applied to population density (PopDen), facility density (Facility), as well as asbestos-cement roof area (ACroof) density and high intervention priority roof area (ACroof_HIP) density (Table 1).

**Table 1:** Neighborhood-level parameter data.

**Index**	**Low Priority**	**Medium Priority**	**High Priority**
Acroof (adim.)	0.17	0.38	0.47
ACroof_HIP (adim.)	0.18	0.44	0.55
PopDen (adim.)	0.18	0.34	0.62
Facility (adim.)	0.18	0.35	0.79
SS (adim.)	0.00	0.50	1.00

Moreover, in Colombia, as in numerous other developing nations, communities are stratified based on socioeconomic status (SS), delineated across six strata ranging from 1 (lowest) to 6 (highest). The socioeconomic strata not only influence roof accessibility but also reflect broader disparities in living conditions, access to resources, and economic opportunities within these communities. In lower SS strata (1–2), households often lack formal ceilings, resulting in direct exposure to asbestos-cement roofs within the living space. Conversely, in higher SS strata (5–6), residences typically feature permanent ceilings that are less accessible to residents. Intermediate SS strata (3–4) often have easily removable ceiling panels, facilitating occasional access to the roof space. Thus, beyond the mere effect on roof access, socioeconomic strata serve as a multifaceted indicator of the socio-economic context, housing infrastructure, and potential exposure pathways to asbestos-containing materials. Accordingly, to quantify the impact of S on asbestos exposure risk, a weighted scoring system is employed, assigning a value of 1.00 to SS strata 1–2, 0.50 to SS strata 3–4, and 0.00 to SS strata 5–6, reflecting the differential vulnerability to asbestos exposure across varying socio-economic contexts.

**Fig. 3:**
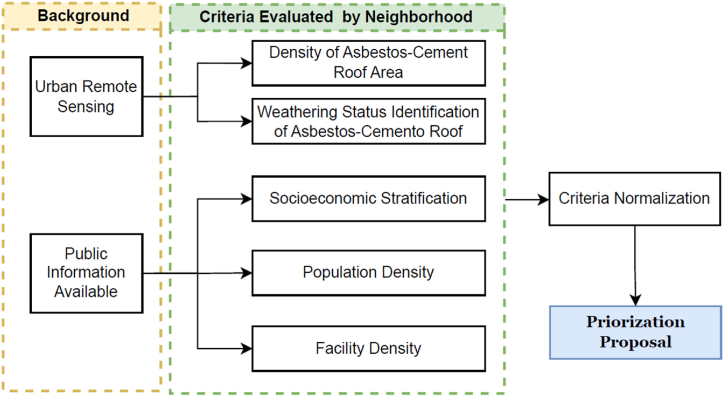
Methodology workflow to establish prioritization of Asbestos-Cement (A) roofs removal.

The aggregation of these six neighborhood-level normalized parameters was accomplished using ArcGIS®, as each parameter is represented by an information layer (Fig. 3).

The outcome of this aggregation (the sum of the 6 criteria) is subsequently normalized within the range of 0–1 to obtain a Priority Intervention Index (PII) at neighborhood-level (see Table 1 with representative data). Ultimately, the PII proposed was discretized in 3 categories, as shown in Table 2, to allow competent authorities to guide public policies and optimize resources.

**Table 2:** Neighborhood-level normalized parameters (representative PII data).

**Priority level**	**PII**
Low (0–0.33)	0.15
Mid (0.34–0.67)	0.39
High (0.68–1)	0.68

## 3 Results and discussion

**Fig. 4:**
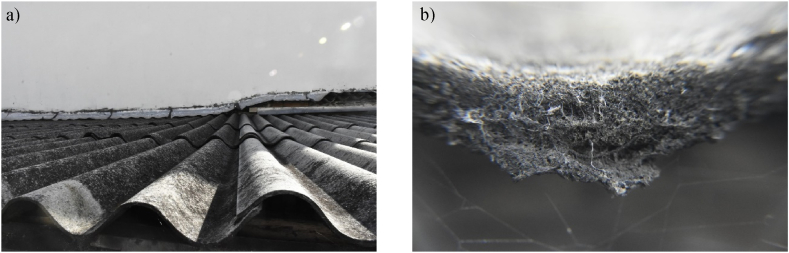
a) Image of a typical AC roof; b) Broken edge showing the chrysotile and crocidolite fibers protruding from the matrix.

**Fig. 5:**
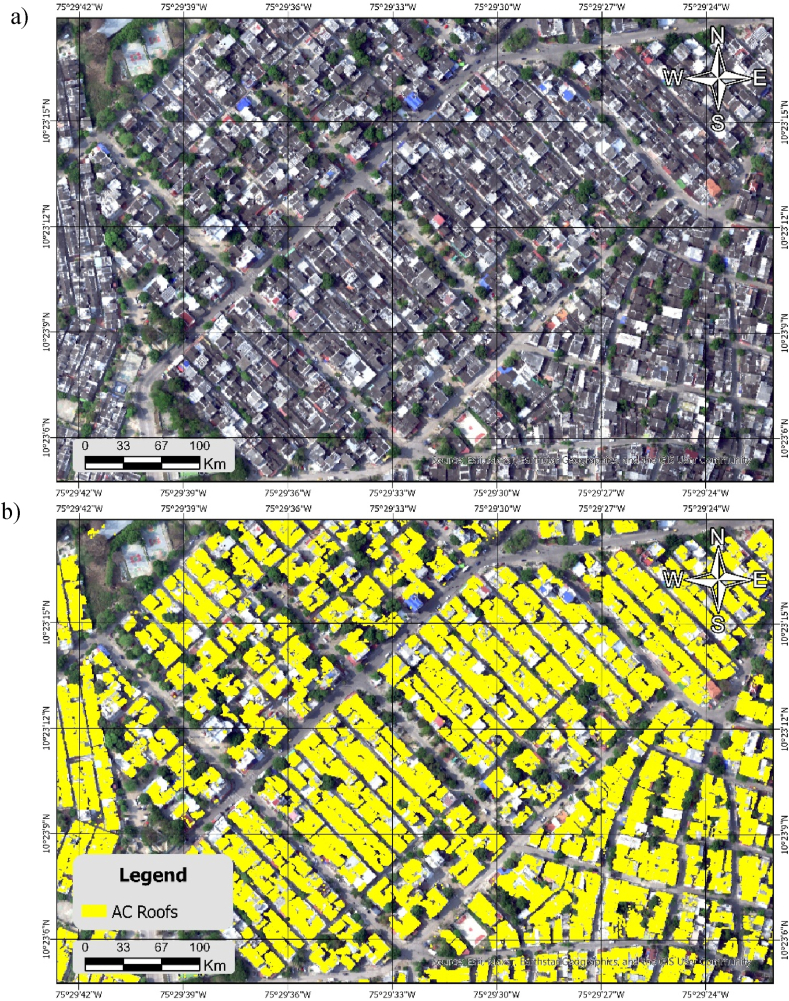
a) Los Caracoles, typical residential neighborhood of Cartagena, one of the 190 neighborhoods studied in the city; b) Asbestos-cement (AC) classification in the neighborhood Los Caracoles.

Polarized light microscopy (PLM) results revealed the presence of asbestos-chrysotile (∼40 % average volume concertation to cement matrix) in all 149 specimens. Crocidolite, another type of asbestos fiber, has been found in 30 % of cases, with average concentrations of 10 %. Fig. 4 a) shows an exemplar asbestos-cement roofing structure, accompanied by a detailed depiction of a fractured section revealing the presence of chrysotile and crocidolite fibers extending from the matrix (Fig. 4 b) (see Fig. 5).

Examination of asbestos-cement using polarized light microscopy (PLM) revealed the presence of crocidolite and chrysotile asbestos fibers in a roof sample. Crocidolite fibers exhibited a distinctive blue-gray color and high relief, while chrysotile fibers appeared nearly invisible with low relief when viewed in a refractive index liquid. Non-fibrous components were identified as carbonates and other minerals commonly found in concrete. Both types of asbestos fibers were abundant, easily detachable, and exhibited a fibrous structure.

### 3.1 AC classification

Fig. 5 (a, b) presents the outcomes of hyperspectral classification applied to asbestos roofs within a segment of the urban centre of Cartagena de Indias. The results underscore the extensive prevalence of asbestos-cement (AC) roofs in a typical residential neighborhood of Cartagena.

**Fig. 6:**
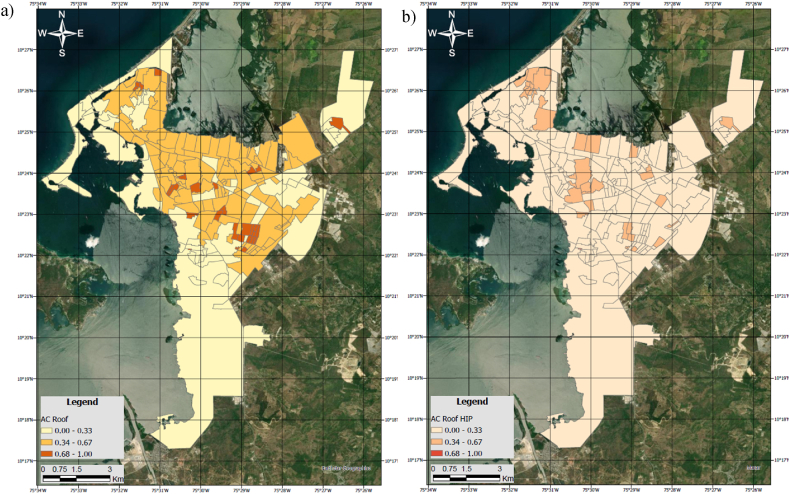
a) Density distribution of the Asbestos-Cement Roof Area (ACroof) across the city at neighborhood level; b) Density distribution of the High Intervention Priority roofs (ACroof_HIP) across the city at neighborhood level. Units are adimensional.

In the 190 distinct neighbourhoods of Cartagena, this study unveiled an average coverage of AC roofs, accounting for 20.4 % of the total neighborhood area, with certain neighbourhoods exhibiting a significant peak of over 47.6 % of their total area being covered by AC roofs (Fig. 6). The cumulative AC roof area identified throughout the city of Cartagena spans a substantial 9.1 square kilometres, a finding that warrants heightened concern among regulatory authorities, academic circles, and the general populace.

The classification model's performance was assessed by comparing its output to ground truth data. The model correctly identified 98 % of asbestos-cement roofs, demonstrating high accuracy. However, the overall accuracy was 94 %, indicating some errors in classifying both asbestos-cement and non-asbestos-cement roofs. These results are consistent with previous studies, but those studies often focused on smaller areas and had fewer ground truth samples. Additionally, there is limited research on densely populated urban areas in developing countries, where buildings are typically smaller and present unique challenges, [23,[25], [26], [27], [28],^40^,^41^].

Furthermore, the map illustrates the density distribution of Asbestos-Cement (AC) Roof Area (Fig. 6 a) and High Intervention Priority (HIP) AC roofs (Fig. 6 b) across the city at the neighborhood level, revealing a prevalent concentration of AC roofs, particularly in the center-north and center-east areas. Notably, 18 % of the city's neighborhoods exhibit a moderate presence of HIP, with the quality of roofs demonstrating a homogeneous distribution throughout.

In the early 1900s, Cartagena's buildings typically had wooden or clay ceilings, emblematic of the prevailing societal norms of the era. Subsequently, during the latter half of the 19th century, a transformative shift occurred with the widespread introduction of zinc and asbestos-cement roofing to the market [42]. Zinc roofs, owing to their cost-effectiveness, gained popularity among the lower social strata, albeit at the compromise of thermal comfort. In contrast, asbestos-cement roofs, offering an optimal cost-benefit ratio, found favor, particularly among middle-income households.

The subsequent decades, spanning from the 1970s to the 1990s, witnessed a noteworthy decline in fiber cement prices. This economic shift facilitated many households in the lower income bracket to transition from zinc roofs to asbestos-cement roofs. Consequently, social strata 3 and 4 presently exhibit the highest density of asbestos-cement covers, followed by strata 1 and 2. In contrast, the more affluent social strata 5 and 6, endowed with greater purchasing power, have migrated towards aesthetically superior and thermally efficient roofing options [43]. This historical trajectory not only underscores the dynamic nature of roofing materials but also reflects the socioeconomic nuances influencing housing choices across different strata in Cartagena.

### 3.2 Priority level criteria

Prioritizing asbestos-cement roof remediation is crucial for regulatory agencies and governments, especially in developing countries or those phasing out asbestos. This is particularly important when there is a significant amount of asbestos but limited resources to remove it. Prioritizing remediation helps maximize outcomes.

**Fig. 7:**
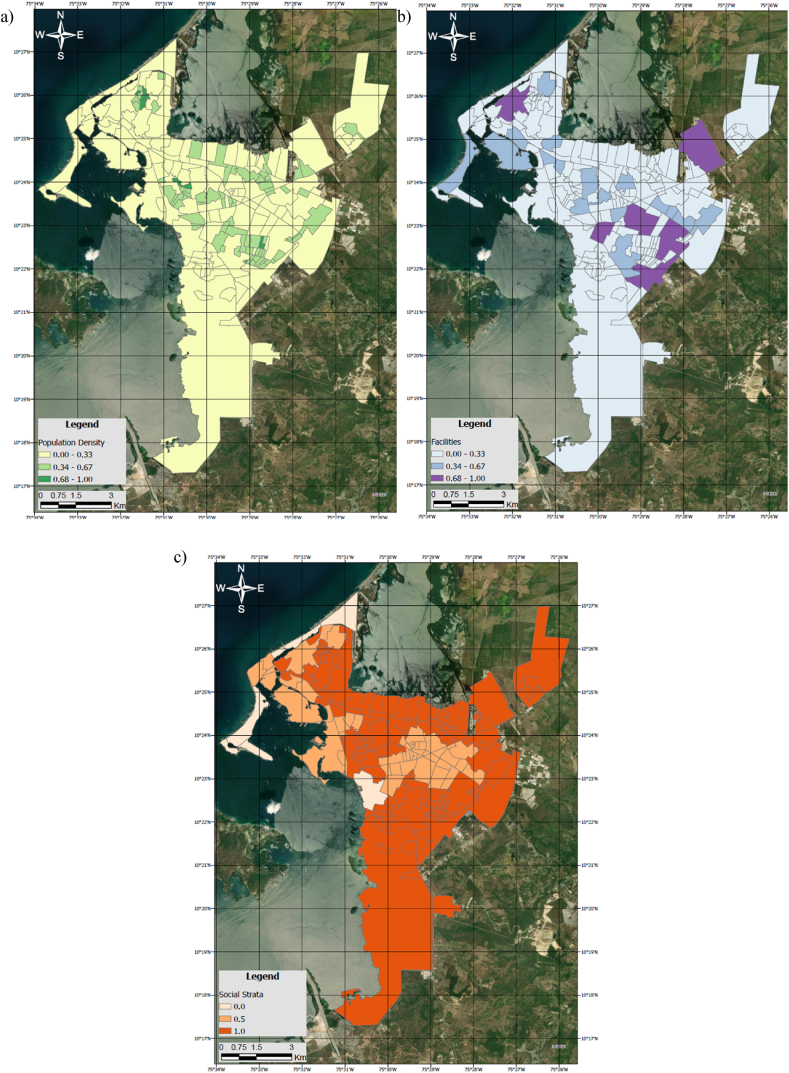
a) Density distribution of the Population (PopDen) across the city at neighborhood level; b) Density distribution of Facilities (Facility) across the city at neighborhood level; c) Density distribution of the Social Strata (SS) across the city at neighborhood level. Units are adimensional.

Fig. 7(a–c) shows the distribution map for population density (PopDen), facility density (Facility) and social strata (SS).

Upon careful analysis of the generated maps, noteworthy observations emerge. The city exhibits a pronounced prevalence of neighborhoods associated with a lower social stratum. An apparent trend of low service density permeates the urban landscape, punctuated by select regions showcasing a concentration of services. Intriguingly, these service-rich areas frequently align with neighborhoods falling within stratum 3–4 and 5–6.

**Fig. 8:**
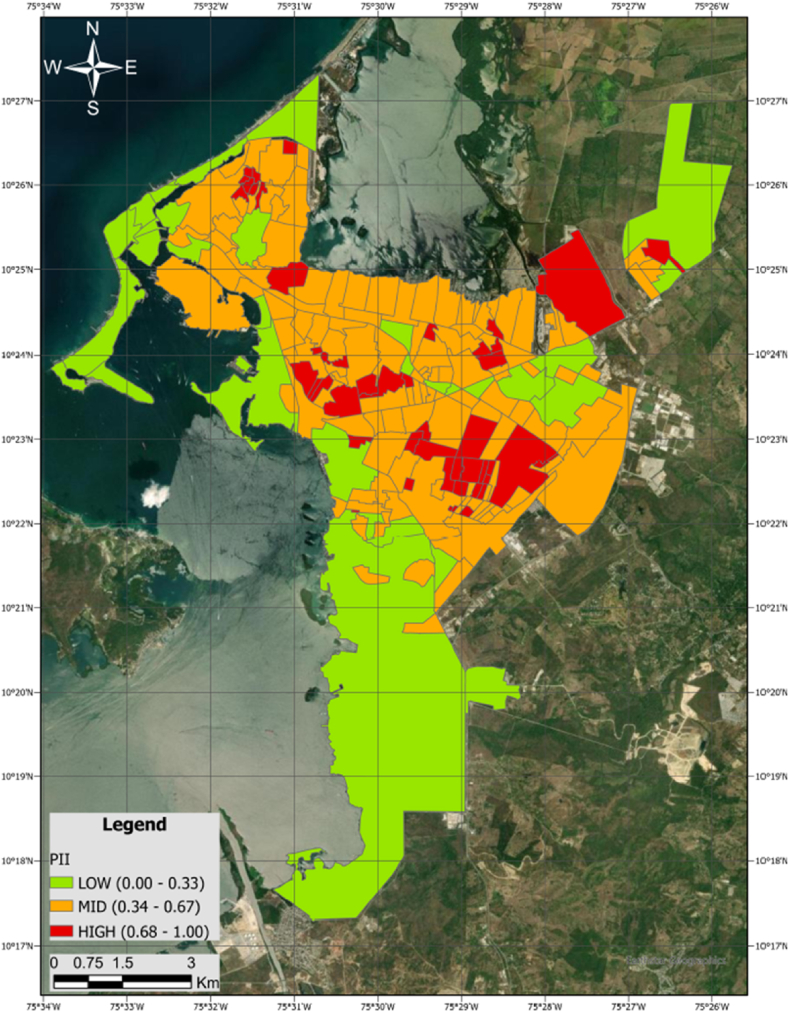
Priority Intervention Index (PII) at neighborhood-level in the case study.

Fig. 8 reveals that areas with the highest priority for intervention align consistently with neighborhoods characterized by social strata 1 and 2. Conversely, neighborhoods falling within social stratum 5–6, along with the industrial zone, exhibit a lower priority for intervention. This stratified approach suggests nuanced patterns in prioritization across different social contexts within the city.

Looking ahead, future studies should consider incorporating variables related to lung health status. Specifically, assessing the densities of Acute Respiratory Infections (ARI), gauging occupational exposure to asbestos, and exploring family histories of asbestos-related diseases would contribute valuable insights. This holistic approach would enhance the comprehensiveness of interventions, incorporating health-related factors for a more nuanced understanding of the impact of asbestos in the studied environment.

The dataset utilized to populate Table 1 encompasses information pertaining to all 190 neighbourhoods, presented in both raw unprocessed format and as normalized parameters. The authors are prepared to furnish these comprehensive datasets to the relevant authorities in Cartagena to aid in the effective management and oversight of asbestos-cement roofs within the region. Such provision of data serves to facilitate informed decision-making and proactive measures regarding asbestos-related concerns in urban settings. A statistical analysis of the data is presented in Annex 1.

The available literature [25,28] for comparative analysis is notably limited, as previous endeavors primarily focus on prioritization exclusively tied to the extent of Asbestos-Cement (AC) coverage, neglecting the incorporation of broader factors, such as demographic and socio-economic considerations. The prevailing literature contemplates the theoretical integration of these multifaceted aspects; however, it lacks a specific prioritization framework that encompasses this comprehensive set of variables. The dearth of comprehensive methodologies in the existing body of work underscores the novelty and significance of the present study, which integrates a nuanced set of criteria to advance the understanding and prioritization of asbestos-related interventions.

## 4 Conclusions

The study aimed to develop a structured, data-based method for determining which asbestos-cement roofs should be repaired first. The proposed methodology aims to overcome the limitations of existing studies and previous authors research by incorporating a diverse set of criteria, resulting in a comprehensive Priority Intervention Index (PII). The main purpose is to offer decision-makers a standardized and transparent tool for assessing and communicating the urgency of interventions. This, in turn, is intended to optimize resource allocation and guide effective public policies to mitigate the risks associated with asbestos exposure in urban environments, particularly in developing countries.

Within the case study of Cartagena de Indias, the ubiquity of asbestos-cement roofing is pervasive, constituting, on average, more than 20 % of the neighborhood's surface area, with peaks approaching 50 %. This prevalence underscores a troubling reality, eliciting apprehension within both academic and societal spheres.

Furthermore, an insightful examination reveals that social stratum emerges as a pivotal factor with substantial weight in the prioritization process. In lower and middle strata, intrinsic and historical factors converge to yield a heightened density of asbestos-cement roofs. These strata, characterized by easy accessibility and high population density, find themselves grappling with a dual challenge—both a higher exposure to this toxic material and socioeconomic vulnerability. Contrastingly, in upper social strata, the principal factor influencing prioritization stems from the heightened concentration of facilities, emphasizing the multifaceted nature of considerations in the strategic management of asbestos-related interventions. This nuanced analysis underscores the intricate interplay of sociodemographic factors and infrastructural dynamics in shaping the prioritization landscape in Cartagena.

## Funding

This research was funded by General System of Royalties of Colombia (10.13039/501100013409SGR, for its initials in Spanish) grant number BPIN 2020000100366.

## Institutional review board statement

Not applicable.

## Informed consent statement

Not applicable.

## Data availability statement

Data will be made available on request.

## CRediT authorship contribution statement

**Leydy K.Torres Gil:** Writing – review & editing, Visualization, Resources, Investigation, Formal analysis, Data curation, Conceptualization. **David Valdelamar Martínez:** Writing – review & editing, Visualization, Resources, Investigation, Formal analysis, Data curation, Conceptualization. **Kellys Babilonia Franco:** Visualization. **Alfonso Arrieta Pastrana:** Funding acquisition. **Manuel Saba:** Writing – review & editing, Writing – original draft, Supervision, Project administration, Methodology, Investigation, Funding acquisition, Formal analysis, Conceptualization.

## Declaration of competing interest

The authors declare the following financial interests/personal relationships which may be considered as potential competing interests:Manuel Saba reports financial support, administrative support, article publishing charges, equipment, drugs, or supplies, and travel were provided by Colombian General System of Royalties. If there are other authors, they declare that they have no known competing financial interests or personal relationships that could have appeared to influence the work reported in this paper.

## Figures and Tables

**Fig. 1A:**
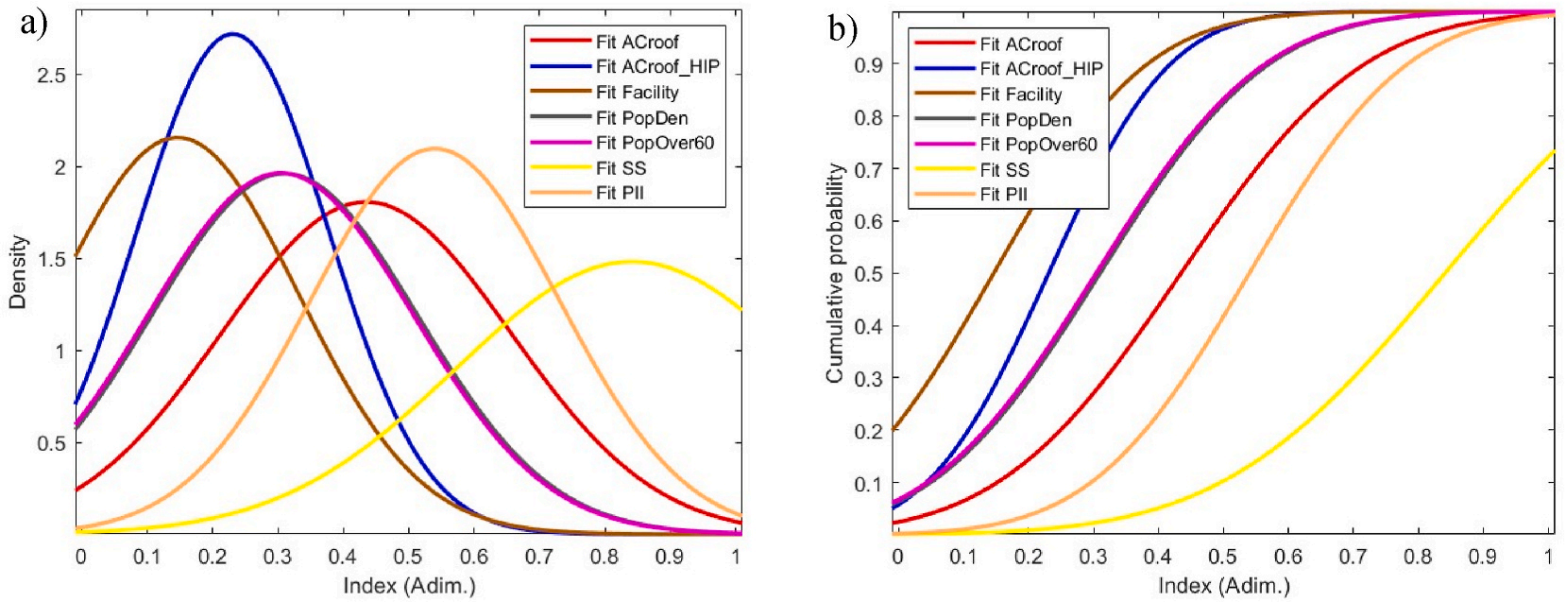
Indexes a) Norma distribution; b) Cumulative distribution.

**Fig. 2A:**
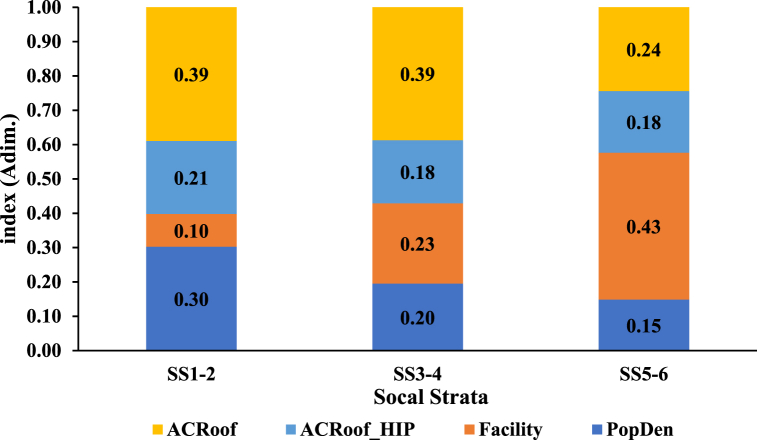
Normalization of indices based on social strata.

## Annex 1

Additionally, a statistical analysis was executed for the indices, aiming to fit each dataset to a probability distribution model. This analytical procedure was carried out utilizing MATLAB R2013b (The MathWorks Inc., Natick, Massachusetts, US), wherein all statistical inferences adhered to a confidence level of 95 % (alpha = 0.05). The adequacy of normal fitted distributions for each index was evaluated through a log likelihood ratio test-based procedure, yielding results within the range of −20 to 98. Fig. 1A a) and b) depict the normal and cumulative distributions of the data.

The obtained data, encompassing indices such as ACroof, ACroof_HIP, Facility, PopDen, SS, and PII, exhibited distinct log-likelihood values, means, and variances. Notably, the analysis of the graphs revealed that the normal distribution aligns well with the asbestos cement roof index (ACRoof) and the Priority Intervention Index (PII).

It is crucial to highlight that the normal distribution did not exhibit a good fit for the Facility, ACroof_HIP, and SS indices. This disparity can be explained by the urban and social dynamics unique to the city, typical of developing nations. In these regions, a substantial proportion (70 %) of neighbourhoods experiences low social status, leading to a shortage of services throughout much of the urban area, often in suboptimal conditions.

Furthermore, Fig. 2A underscores a discernible trend, a decrease in population density corresponding to an upward shift in the social stratum, accompanied by a higher prevalence of facilities and a reduced prevalence of asbestos-cement roofs.
